# Supplementary material for: Enhanced Response for Foodborne Pathogens Detection by Au Nanoparticles Decorated ZnO Nanosheets Gas Sensor
Source: Biosensors (Basel). 2022 Sep 28;12(10):803. doi: 10.3390/bios12100803 (PMC9599186; doi:10.3390/bios12100803)
Supplement: Supplementary file 1 [file biosensors-12-00803-s001.zip › biosensors-1919264-supplementary.pdf]

biosensors

MDPI

Supplementary Material

Enhanced Response for Foodborne Pathogens Detection by Au Nanoparticles Decorated ZnO Nanosheets Gas Sensor

Cheng Zhao [1], Shanshan Xu [2], Jing Wei [3], Siqi Xie [1], Jinlei Wei [1], Jingting Han [1], Zhaohuan Zhang [1], Haiquan Liu [1], Jinsheng Cheng [4], Yong Zhao [1,*] and Yongheng Zhu [1,*]

[1] College of Food Science and Technology, Shanghai Ocean University, Shanghai 201306, China
[2] State Key Laboratory of Chemical Engineering, College of Chemical and Biological Engineering, Zhejiang University, Hangzhou 310027, China
[3] The Key Laboratory of Biomedical Information Engineering of Ministry of Education, the Institute of Analytical Chemistry and Instrument for Life Science, School of Life Science and Technology, Xi'an Jiaotong University, Xi'an 710049, China
[4] Henry−Fork School of Food Sciences, Shaoguan University, Shaoguan 512005, China
* Correspondence: yzhao@shou.edu.cn (Yong Zhao); yh-zhu@shou.edu.cn (Yongheng Zhu); Tel.: +86-15692165928 (Yong Zhao); +86-15000137862 (Yongheng Zhu)

Preparation of ZnO sensors

The microelectron mechanical systems (MEMS) sensor possesses interdigital electrode and an integrated micro heater, as shown in Figure S1. Specifically, the resistance changes can be observed by interdigital electrode, and stable working temperature can be provided by microheater.

The ZnO sensors was prepared according to the previous research [27]. First of all, the gas-sensitive material and ethanol were transferred into an agate mortar with polishing for a few minutes to obtain a paste. Secondly, a drop of above sample was added to the Pt interdigital electrode. After drying in infrared drying oven, the ZnO sensors were aged at 230 °C.

The testing process and the results were displayed through an intelligent analysis system. The working temperature of material chip was regulated by changing the heating voltage. During the test, an accurately calculated target liquid was injected onto a heated plate with a micro syringe, where the fan helped the injected liquid to vaporize rapidly. It can be visualized that the load resistance (R_L) and the MEMS sensor formed a series circuit (Figure S1b), and at the same time the output voltage (V_{out}) could be recorded by software automatically.

Gas sensor measurement

The gas sensing performance of the gas sensor was measured by a static test system which records real-time change in resistance of sensor. The different concentrations of vapors were obtained by injecting liquid or gas of volume Q into a testing chamber. The volume Q can be determined by **Eq 1**:

$$Q = \frac{V \times C \times M}{22.4 \times d \times \rho} \times 10^{-9} \times \frac{273+T_R}{273+T_B} \quad (1)$$

Here, V, C, M, d, ρ, T_R, and T_B are the test chamber volume (90 mL), vapor concentration (ppm), molecular mass, liquid density, liquid purity, environmental temperature, and temperature in the testing chamber, respectively. 3-hydroxy-2-butanone (3H-2B) vapor was obtained by evaporating 8% 3H-2B solution. For the reducing gases and n-type MOS, the response of gas sensors is defined as S = R_a/R_g, where R_a is the gas sensor's resistance in air atmosphere and R_g is the gas sensor's resistance in target gas atmosphere. The response and recovery time are defined as the time taken by the gas sensor to achieve the resistance changes ranging from R_a to R_a-90% (R_a-R_g) and from R_g to R_g+90% (R_a-R_g) in the case of adsorption and desorption of target gases, respectively. And the relationship between the resistance (R) and the voltage (V_{out}) are listed in **Eq2**:

$$R = \frac{(5-V_{out})}{V_{out}} \times R_L \quad (2)$$

In which, the circuit voltage (V) and the load resistance (R_L) were set at 5 V and 100 KΩ, respectively. And at the same time the output voltage (V_{out}) could be recorded by software automatically.

Figure and Table caption:

Figure S1. Schematic illustration for the description of MOSs sensors. **(a)** The panoramic view of the MEMS gas sensor test system. **(b)** The measuring circuit of MEMS gas sensor. **(c)** The test base schematic of the MEMS gas sensor. **(d)** The exploded views of test base.

Figure S2. The N_2 adsorption-desorption isotherms and BJH pore size distribution of **(a)** ZnO NS and **(b)** 0.5% Au/ZnO NS.

Figure S3. The detection limit of 1.0% Au/ZnO NS towards 0.5 ppm 3H-2B at 230 °C.

Figure S4. The long-term stability of 0.5% Au/ZnO NS, 1.0% Au/ZnO NS, 1.5% Au/ZnO NS, 2.0% Au/ZnO NS towards 10 ppm 3H-2B at 230 °C.

Table S1. The comparison of the gas sensing properties of 1.0% Au/ZnO NS sensors with other MOS-based 3H-2B sensors in the previous researches.

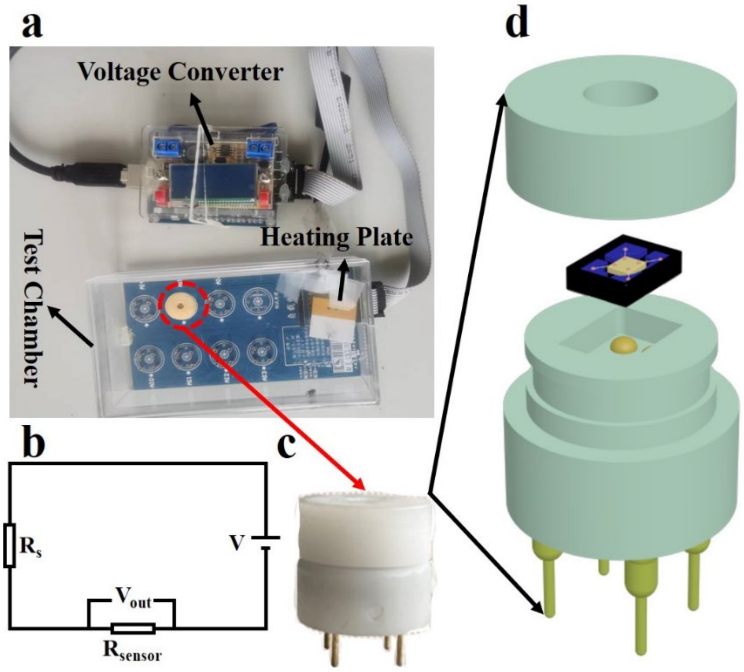

Figure S1. Schematic illustration for the description of MOSs sensors. **(a)** The panoramic view of the MEMS gas sensor test system. **(b)** The measuring circuit of MEMS gas sensor. **(c)** The test base schematic of the MEMS gas sensor. **(d)** The exploded views of test base.

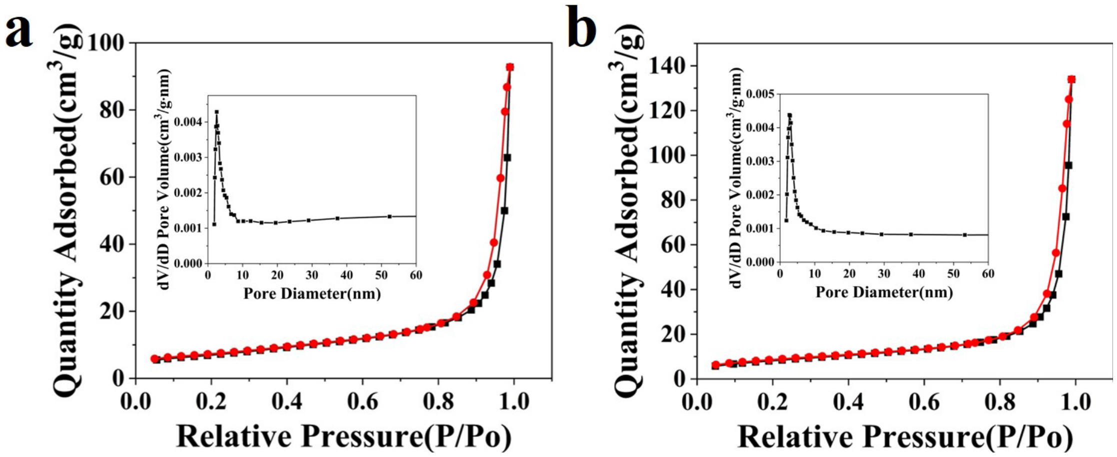

Figure S2. The N_2 adsorption-desorption isotherms and BJH pore size distribution of **(a)** ZnO NS and **(b)** 0.5% Au/ZnO NS.

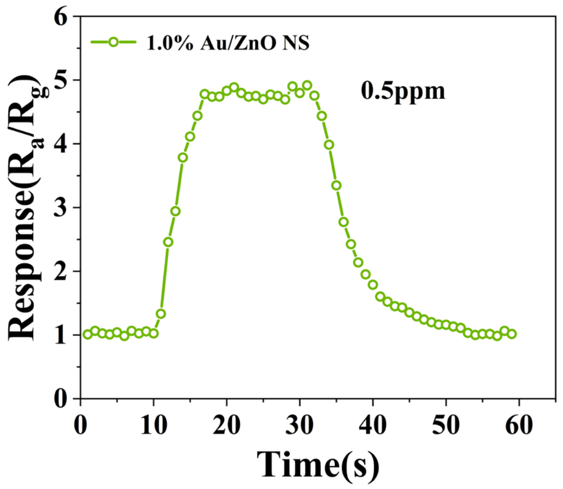

Figure S3. The detection limit of 1.0% Au/ZnO NS towards 0.5 ppm 3H-2B at 230 °C.

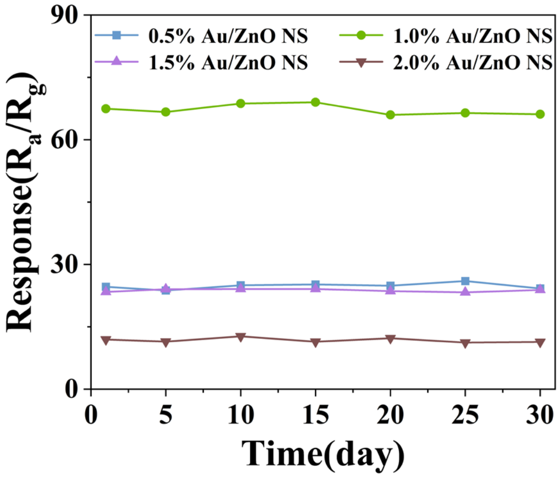

Figure S4. The long-term stability of 0.5% Au/ZnO NS, 1.0% Au/ZnO NS, 1.5% Au/ZnO NS, 2.0% Au/ZnO NS towards 10 ppm 3H-2B at 230 °C.

Table S1. The comparison of the gas sensing properties of 1.0% Au/ZnO NS sensors with other MOS-based 3H-2B sensors in the previous researches.

$T_{owt.}$ = optimum working temperature, 3H-2B conc. = concentration of 3H-2B gas, LOD = limit of detection.

Sensing materials	$T_{owt.}$ (°C)	3H-2B conc. (ppm)	S (R_a/R_g)	Response-recovery time	LOD (ppm)	Refs
SnO_2-Al_2O_3 nanocables	120	5	43.3	48/194	0.1	[10]
Mesoporous WO_3	290	25	56.1	4/13	0.1	[7]
Cr/WO_3 nanofibers	140	5	71.8	21/36	0.05	[5]
Pt/SnO_2 mesoporous hollow nanospheres	250	10	48.68	11/22	0.5	[8]
Pd/$BiVO_4$ decahedrons	200	10	103.7	12/8	0.2	[36]
ZnO-Al_2O_3 nanocables	300	50	37.2	27/34	1	[11]
Hydrangea-like mesoporous WO_3 nanoflowers	205	25	152	25/146	0.4	[9]
Au/ZnO nanosheets	230	25	174.04	6/7	0.5	This work

References **(The order numbers of references are consistent with that in the Manuscript)**

5. Zhu, Z.Y.; Zheng, L.J.; Zheng, S.Z.; Chen, J.; Liang, M.H.; Tian, Y.T.; Yang, D.C. Cr doped WO_3 nanofibers enriched with surface oxygen vacancies for highly sensitive detection of the 3-hydroxy-2-butanone biomarker. *J. Mater. Chem. A* **2018**, *6*, 21419–21427.

7. Zhu, Y.H.; Zhao, Y.; Ma, J.H.; Cheng, X.W.; Xie, J.; Xu, P.C.; Liu, H.Q.; Liu, H.P.; Zhang, H.J.; Wu, M.H.; Elzatahry, A.A.; Alghamdi, A.; Deng, Y.H.; Zhao, D.Y. Mesoporous tungsten oxides with crystalline framework for highly sensitive and selective. *J. Am. Chem. Soc.* **2017**, *139*, 10365–10373.

8. Cai, H.J.; Liu, H.Q.; Ni, T.J.; Pan, Y.J.; Zhao, Y.; Zhu, Y.H. Controlled synthesis of Pt doped SnO2 mesoporous hollow nanospheres for highly selective and rapidly detection of 3-hydroxy-2-butanone biomarker. *Front. Chem.* **2019**, *7*, 843.

9. Xu, D.P.; Ge, K.J.; Qi, S.Y.; Chen, Y.; Qiu, J.X.; Liu, Q. Hydrangea-like mesoporous WO_3 nanoflowers with crystalline framework for 3-hydroxy-2-butanone sensing. *Anal. Bioanal. Chem.* **2020**, *412*, 8371–8378.

10. Xing, X.X.; Zhu, Z.Y.; Du, L.L.; Feng, D.L.; Chen, J.; Li, S.; Yang, D.C. Burrs-shelled SnO2@Al2O3 nanocables for detection of 3-hydroxy-2-butanone biomarkers. *Appl. Surf. Sci.* **2020**, *502*, 144106.

11. Chen, J.; Zhu, Z.Y.; Zheng, S.Z.; Du, L.L.; Xing, X.X.; Feng, D.L.; Li, S.; Yang, D.C. Synthesis of zinc oxide-alumina nanocables for detection of 3-hydroxy-2-butanone biomarker. *Mater. Lett.* **2019**, *253*, 121–123.

27. Shen, J.B.; Xu, S.S.; Zhao, C.; Qiao, X.P.; Liu, H.Q.; Zhao, Y.; Wei, J.; Zhu, Y.H. Bimetallic Au@Pt nanocrystal sensitization mesoporous alpha-Fe_2O_3 hollow nanocubes for highly sensitive and rapid detection of fish freshness at low temperature. *ACS Appl. Mater. Inter.* **2021**, *13*, 57597–57608.

36. Chen, J.; Feng, D.L.; Wang, C.; Xing, X.X.; Du, L.L.; Zhu, Z.Y.; Huang, X.H.; Yang, D.C. Gas sensor detecting 3-hydroxy-2-butanone biomarkers: Boosted response via decorating Pd nanoparticles onto the {010} facets of $BiVO_4$ decahedrons. *ACS Sens.* **2020**, *5*, 2620–2627.
